# Herbal extract fermented with inherent microbiota improves intestinal health by exerting antioxidant and anti-inflammatory effects in vitro and in vivo

**DOI:** 10.1186/s40104-025-01178-w

**Published:** 2025-04-06

**Authors:** Mara Heckmann, Nadiia Sadova, Georg Sandner, Cathrina Neuhauser, Bernhard Blank-Landeshammer, Bettina Schwarzinger, Alice König, Meizhen Liang, Michael Spitzer, Julian Weghuber, Verena Stadlbauer

**Affiliations:** 1 Center of Excellence Food Technology and Nutrition, University of Applied Sciences Upper Austria, Stelzhamerstraße 23, 4600 Wels, Austria; 2 FFoQSI GmbH–Austrian Competence Centre for Feed and Food Quality, Safety and Innovation, Technopark 1D, 3430 Tulln, Austria; 3 TVA Produktions- & Vertriebs-Gesellschaft m.b.H, Dorf 156, 3343 Hollenstein, Austria

**Keywords:** *C. elegans*, *D. melanogaster*, Feed additive, Fermented plant extract, Immune response, Intestinal barrier, Oxidative stress

## Abstract

**Background:**

Maintaining intestinal health is crucial for the overall well-being and productivity of livestock, as it impacts nutrient absorption, immune function, and disease resistance. Oxidative stress and inflammation are key threats to intestinal integrity. This study explored the antioxidant, anti-inflammatory, and barrier-strengthening properties of a fermented plant macerate (FPM) derived from 45 local herbs, using a specifically developed fermentation process utilizing the plants’ inherent microbiota to enhance bioactivity and sustainability.

**Results:**

In vitro experiments with IPEC-J2 cells showed that FPM significantly reduced intracellular reactive oxygen species (ROS) levels, improved barrier integrity, and enhanced cell migration under stress. Similar antioxidant effects were observed in THP-1 macrophages, where FPM reduced ROS production and modulated inflammatory responses by decreasing pro-inflammatory cytokines [tumor necrosis factor alpha (TNF-α), monokine induced by gamma interferon (MIG), interferon-inducible T cell alpha chemoattractant (I-TAC), macrophage inflammatory proteins (MIP)-1α and -1β] and increasing anti-inflammatory interleukin (IL)-10 levels. Mechanistic studies with HEK-Blue reporter cell lines revealed that FPM inhibited nuclear factor kappa B (NF-κB) activation via a toll-like receptor (TLR)4-independent pathway. In vivo, FPM significantly reduced ROS levels in *Drosophila melanogaster* and improved activity and LT_50_ values in *Caenorhabditis elegans* under oxidative stress, although it did not affect intestinal barrier integrity in these models.

**Conclusion:**

The findings indicate that FPM shows promising application as a functional feed supplement for improving intestinal health in livestock by mitigating oxidative stress and inflammation. Further studies, including livestock feeding trials, are recommended to validate these results.

**Supplementary Information:**

The online version contains supplementary material available at 10.1186/s40104-025-01178-w.

## Background

Herbs and herbal mixtures have long been recognized as therapeutic medication, benefiting both human and animal health [1]. These natural products are rich in bioactive molecules, such as flavonoids, terpenoids, and alkaloids, which are known for possible anti-inflammatory, antioxidant, and antimicrobial effects [2]. Incorporating herbal plants into animal diets offers a scientifically grounded approach to enhancing gut health and reproductive performance, reducing inflammation, and improving overall growth and health. This holistic approach not only improves animal welfare but also supports sustainable livestock farming by reducing reliance on synthetic drugs and antibiotics [3].

Oxidative stress and chronic inflammation in livestock, often caused by factors such as microbial infections, suboptimal nutrition, and environmental conditions like heat stress, can impair growth and increase disease susceptibility [4]. Heat stress, in particular, is a growing concern due to global warming, which leads to elevated temperatures that can exacerbate inflammation and oxidative damage [5]. Furthermore, a compromised intestinal barrier can increase permeability, allowing pathogens and toxins to enter the bloodstream, triggering systemic inflammation and disease [6, 7]. Maintaining a robust intestinal barrier is therefore crucial for preventing these adverse effects and ensuring optimal animal health [8]. The use of bioactive compounds from herbs such as curcumin, capsaicin and quercetin can modulate inflammatory pathways, reduce cytokine production, strengthen the mucosal lining and promote the intestinal health of hosts by regulating the structure and abundance of intestinal microbiota and metabolites of bacterial flora [9, 10]. These effects enhance nutrient absorption and improve immune function, contributing to better overall health and resilience in livestock.

However, the generally low bioavailability of natural compounds often limits their therapeutic potential. Many bioactive compounds are poorly absorbed in the gut, rapidly metabolized, and quickly excreted, reducing their effectiveness [11]. To address this challenge, the fermentation of herbal products is emerging as a technique to enhance the absorption and efficacy of the contained compounds [12]. During this process, which involves the action of microorganisms, complex molecules are hydrolyzed into simpler, more bioavailable derivatives, thereby enhancing the solubility and absorption of bioactive reagents and generating novel metabolites with augmented biological activity [13]. Fermentation is becoming increasingly common in the livestock industry as it offers several benefits [14]. Fermentation has been shown to increase the concentration of active ingredients, boost antioxidant properties, and enhance the overall effectiveness of herbal supplements [15]. Supporting these findings, Zhang et al. [16] reported that fermentation significantly improved both the availability and anti-inflammatory activity of bioactive compounds in *Gingko biloba* leaves tested in immune-stressed chickens, highlighting its role in optimizing their therapeutic value. Moreover, a recent meta-analysis demonstrated that fermented herbal products, compared to unfermented herbs, yielded significantly greater improvements in growth performance, breast meat quality, and intestinal morphology in broiler chickens. This enhancement was partially attributed to an increased ratio of villus height to crypt depth in the duodenum and jejunum, thereby expanding the absorptive surface area [17].

In this study, we evaluated the health-promoting effects of a fermented plant macerate (FPM) comprising 45 distinct herbs locally cultivated in the Lower Austria region. Our objective was to utilize local biodiversity and distinctive fermentation techniques to enhance the health benefits of herbal feed supplements while maintaining the integrity and potency of the herbal components and promoting a sustainable production process. We hypothesized that FPM strengthens the intestinal barrier integrity and mitigates oxidative stress and inflammation by inhibiting the production of pro-inflammatory and prooxidant signaling molecules. To investigate the underlying mechanisms of FPM, we implemented and employed several in vitro (IPEC-J2, THP-1 and HEK-Blue cells) and in vivo (*Drosophila melanogaster* and *Caenorhabditis elegans*) models to simulate inflammatory, oxidative and mechanical stress conditions.

## Material and methods

### Reagents under investigation

The investigated FPM *KE-agrar* was obtained from TVA Produktions- & Vertriebs-GmbH (Hollenstein, Austria). It is sold as a feed supplement containing the natural plant microbes of the herbs. The extract was prepared from 45 herbs. The ratio of all 45 herbs in FPM is provided in Additional file 1, Table S1. The herbs were regionally grown, washed, and cut after harvesting. The extract preparation involved a two-stage process. First, the herbs were macerated in drinking water with added mineral salts under alkaline conditions. This macerate was then fermented by adding organic sugar cane molasses, which promoted the growth of the plants’ inherent microbes. The microbial activity lowered the pH to 3.2–3.4, stabilizing the product. The final composition of FPM was 0.025% herbs, 0.01% mineral salts, 3.2% organic sugar cane molasses, and 96.765% drinking water. For this study, all indicated concentrations of FPM used in the experiments are expressed as [v/v].

For analysis of the bioactivity of volatile compounds, 1 mL of FPM were dried in a Vacuum Concentrator (Labconco, Kansas City, USA) at 25 °C for 9 h and the dried residue was resuspended in 1 mL sterile distilled water.

The cytotoxicity of FPM in each cell line was assessed after a 24-h incubation period by treating the cells with a 50 µmol/L resazurin sodium salt solution (Sigma Aldrich, St. Louis, MO, USA). Fluorescence intensity was measured using a POLARstar Omega microplate reader (BMG LABTECH, Ortenberg, Germany) with excitation and emission wavelengths of 544 nm and 590 nm, respectively. Only non-toxic concentrations of FPM (cell viability > 90% of control) were used in all subsequent cell culture experiments.

### Cell culture and differentiation

Intestinal porcine epithelial cells-jejunum 2 (IPEC-J2) (ACC 701) were purchased from DSMZ (Braunschweig, Germany) and human Tohoku Hospital Pediatrics-1 (THP-1) cells (TIB-202) were obtained from ATCC (Manassas, VA, USA). The cells were maintained in Dulbecco’s Modified Eagle Medium (DMEM, IPEC-J2) or and Roswell Park Memorial Institute 1640 Medium (RPMI 1640, THP-1) supplemented with 10% fetal bovine serum (FBS) and 100 U/mL penicillin/100 μg/mL streptomycin (all PAN-Biotech, Aidenbach, Germany) and grown at 37 °C in a humidified atmosphere (≥ 95%) with 5% CO_2_. THP-1 cells were differentiated from monocytes in suspension to adherent macrophages for all experiments. The cells were seeded in growth medium supplemented with 50 ng/mL phorbol 12-myristate 13-acetate (PMA, Sigma-Aldrich) and grown for 48 h until the differentiation process was completed. Successful differentiation was defined by an adherence rate of ~90%. The HEK-Blue hTLR4 reporter cell line, which stably co-expresses a nuclear factor kappa B (NF-κB)-inducible secreted embryonic alkaline phosphatase (SEAP) reporter gene and the human toll like receptor 4 (*TLR4*) gene, was obtained from InvivoGen (San Diego, CA, USA), along with its corresponding parental cell line, HEK-Blue Null2, which only expresses the SEAP reporter gene. These cell lines were cultured at 37 °C in a 5% CO_2_ and humidified air atmosphere (≥ 95%) using DMEM supplemented with 10% heat-inactivated FBS, 100 U/mL penicillin, 100 μg/mL streptomycin, and 100 μg/mL Normocin™ (InvivoGen). Additionally, selective antibiotics 1X HEK-Blue Selection were used for the HEK-Blue hTLR4 cell line, and 100 μg/mL Zeocin for the HEK-Blue Null2 cell line (both from InvivoGen).

### Determination of intracellular reactive oxygen species

For intracellular reactive oxygen species (ROS) measurements, IPEC-J2 cells were seeded in black 96‐well plates at 2 × 10^4^ cells per well and grown overnight under standard conditions. THP-1 cells were seeded at 1 × 10^5^ cells per well and differentiated as described above. On the following day, the cells were washed twice with Dulbecco’s phosphate-buffered saline (DPBS, PAN-Biotech) followed by a 20 min co-treatment with the antioxidant control quercetin (QUE, 20 µmol/L, Sigma-Aldrich) or FPM in combination with 50 µmol/L of the ROS probe 2´,7´-dichlorodihydrofluorescein diacetate (H_2_DCFDA, Sigma-Aldrich) in Hank’s balanced salt solution (HBSS, PAN-Biotech). This procedure was described previously [18, 19]. Oxidative stress was induced with 500 µmol/L 2,2´-azobis(2-methylpropionamidine) dihydrochloride (AAPH, Sigma-Aldrich). The amount of 2´,7´-dichlorodihydrofluorescein (H_2_DCF), the oxidized form of H_2_DCFDA, was determined with the POLARstar Omega microplate reader in fluorescence mode at 485 nm excitation and 520 nm emission. The measurements were performed immediately after stressor addition and after 15, 30, 60 and 90 min. The oxidative status over this period was summarized by calculation of the area under the curve (AUC) and normalized to the AAPH stress control.

### Intestinal barrier analysis in vitro

In vitro intestinal barrier integrity was analyzed by a cell culture insert‐based assay with IPEC-J2 cells, as described previously with slight modifications [20]. In detail, 3 × 10^4^ IPEC-J2 cells were seeded per insert (ThinCert, 0.336 cm^2^, 0.4 μm pore diameter, Greiner-Bio One, Kremsmünster, Austria) to reach confluence on the next day. The cells were further maintained in full growth medium which was exchanged every 2–3 d. Trans-epithelial electrical resistance (TEER) values were measured before each medium exchange during differentiation with a Millicell‐ERS‐2 Voltohmmeter (Merck, Darmstadt, Germany). When the values reached levels higher than 1,000 Ωcm^2^ 5 d after seeding, the assay was started. The cells were treated with 1% and 2% FPM in full growth medium for 48 h and it was renewed after 24 h. Subsequently, oxidative stress was induced by addition of 250 µmol/L tert-butyl hydroperoxide (tBHP, Sigma-Aldrich). In inserts with cells pretreated with FPM, the same concentrations of FPM were added with the tBHP. The intestinal barrier integrity during oxidative stress was analyzed immediately and 2, 4, 6, 24 and 48 h after tBHP addition by the detection of the TEER values.

### Cell migration analysis

IPEC-J2 cells were seeded in 24‐well imaging plates (MoBiTec, Göttingen, Germany) at 2 × 10^5^ cells per well and grown overnight. Then, a straight scratch was generated with constant pressure, speed and angle using a 100 μL plastic pipette tip. Excess debris was removed by washing with DPBS and replaced by full growth medium with or without FPM. For scanning of the scratch wounded area, automated brightfield imaging was performed every 15 min for a total of 315 min on an epi-fluorescence microscope (Nikon Eclipse Ti2, Nikon, Tokyo, Japan) using a 2× CFI Plan Apochromat objective (NA = 0.1) with additional 1.5× magnification. The microscope was further equipped with a cage incubator (Okolab, Shanghai, China) for temperature and CO_2_ control, a Zyla 4.2 sCMOS camera (Andor, Oxford Instruments, Abingdon, UK), and an x-y-stage (CMR-STG-MHIX2-motorized table, Märzhäuser Wetzlar, Wetzlar, Germany) allowing scanning of multiple stage positions in one experiment. Cage incubator preheating and CO_2_ equilibration was started 1 h prior to experiment and illumination adjustment was performed after the plate was inserted to the microscope stage. Acquisition positions were defined under the control of the NIS-Elements software package (Version 5.02.01, Nikon) with respect to the scratch in each individual well. Image analysis was carried out using *ImageJ* and the “*Wound_healing_size_tool.ijm*” plugin [21]. Cell front velocity (μm/h) was calculated as indicated:

$$\mathrm{Cell}\;\mathrm{front}\;\mathrm{velocity}=\frac{\mathrm{wound}\;\mathrm{closure}\;\mathrm{speed}}{\mathrm{length}\;\mathrm{of}\;\mathrm{cell}\;\mathrm{front}\;\times\;2}$$

with *wound closure speed* calculated from the slope of the linear trend line (15−120 min) and the *length of cell front *$\times$ 2 corresponds to the length of the gap area considering two cell fronts that migrate towards each other (see also “ibidi- cells in focus”).

### Cytokine secretion analysis

THP-1 cells were seeded in 6‐well plates at 2.75 × 10^6^ cells per well and differentiated as described above. Subsequently, the cells were stressed with 250 ng/mL lipopolysaccharides from *E. coli* (LPS, Sigma-Aldrich) with or without 0.5% or 1% FPM for 24 h. For the treatment, medium with a reduced serum concentration of 1% FBS was used.

The membrane-based human cytokine antibody array (Proteome Profiler Human XL Cytokine Array Kit, Bio-Techne, Minneapolis, MN, USA) was performed according to manufacturer’s instructions using 350 µL of each cell culture supernatant sample. For imaging of the membranes, a ChemiDoc MP System (Bio-Rad Laboratories, Hercules, CA, USA) was used. Image analysis of signal intensity was performed with Image Lab Version 6.0.0 (Bio-Rad Laboratories).

The multiplex immunoassay (7-plex Discovery Assay, Bio-Techne) was performed according to manufacturer’s instructions with the Luminex® 200 RUO System w/xPONENT 4.2 (Luminex Corporation, Austin, TX, USA) using undiluted and 1:100 diluted samples. The 7-plex Assay included the analytes for the detection of necrosis factor alpha (TNF-α), monokine induced by gamma interferon (MIG), interferon-inducible T-cell alpha chemoattractant (I-TAC), macrophage inflammatory protein (MIP)-1α, MIP-1β, interleukin (IL)-10 and IL-27.

### NF-κB activation analysis

To assess NF-κB activation after treatment with LPS, TNF-α, and FPM, the HEK-Blue hTLR4 reporter cell line and its parental cell line HEK-Blue Null2 were used by measuring the amount of secreted SEAP, a downstream target. The use of the parental cell line helped to determine the involvement of the TLR4 receptor. Briefly, 20 µL of LPS, TNF-α, and FPM, with or without LPS and TNF-α, diluted in DMEM (supplemented with 10% heat-inactivated FBS, 100 U/mL penicillin, 100 μg/mL streptomycin, and 100 µg/mL Normocin™) to final concentrations of 10 ng/mL LPS or TNFα and 0.01%, 0.025%, or 0.05% FPM, were added to 96-well plates. Then, 180 µL of HEK-Blue hTLR4 cells and HEK-Blue Null2 cells were seeded in triplicate into the plates containing the treatment solutions at densities of 1 × 10^5^ and 0.6 × 10^5^ cells per well, respectively. After incubating for 24 h at 37 °C, 20 µL of the cell culture supernatants were mixed with 180 µL of QUANTI-Blue (InvivoGen) solution containing the SEAP substrate. Absorbance was measured at 620 nm after 30 min of incubation at 37 °C using the POLARstar Omega microplate reader. Cell viability was confirmed using the CellTiter-Glo® Cell Viability Assay (Promega Corporation, Madison, WI, USA) according to the manufacturer’s instructions. Results were corrected for background and normalized to the stress controls.

### Cultivation and age synchronization of *Caenorhabditis elegans*

*C. elegans* strains were cultivated as previously described [22] and maintained at 20 °C or 15 °C. All strains [wild-type Bristol (N2), *glp-4*(bn2) (SS104)] and the bacterial food source (OP50) were obtained from the *C. elegans* Genetics Center (University of Minnesota, MN, USA).

To synchronize nematode populations, gravid worms were washed off plates with M9-PEG buffer (M9 buffer containing 0.5% w/v polyethylenglycol PEG 3.350) [22], and residual bacteria were removed by washing worms once with M9-PEG buffer prior to bleaching gravid adults using 1% (v/v) alkaline hypochlorite solution to obtain eggs [22]. Nematodes dissolved after ~5 min at room temperature under constant shaking. Hypochlorite was removed by washing the eggs two times with M9-PEG buffer. Eggs were transferred onto fresh OP50 seeded nematode growth medium (NGM) control or treatment plates and were further incubated to the desired development stage.

### Oxidative stress analysis in *C. elegans*

To evaluate protective effects of FPM under oxidative stress, an infrared micro-beam-based system was utilized to record locomotor activity over time. After 72 h treatment with FPM, adult N2 nematodes were washed off plates using S-Basal (+0.5% w/v PEG 3.350) [22]. The number of nematodes was adjusted with S-Basal to reach approximately 80 worms per well. Next, 92.5 µL worms in S-Basal were pipetted into individual wells of a flat-bottom 96-well plate. The experiment was performed in eight well replicates per testing group. Basal nematode activity was measured for 60 min using WMicrotracker One (Phylumtech, Santa Fe, Argentina) to obtain reference values for individual well data normalization. Next, oxidative stress was induced by adding 7.5 µL of 1 mol/L paraquat (PQ) in dH_2_O stock solution resulting in 75 mmol/L final concentration per well. For non-stressed groups, dH_2_O was added instead of PQ. Nematode activity was captured for a total duration of 1,000 min using WMicrotracker One. Results are given as locomotive motility normalized to basal activity. For AUC calculation, data was normalized to the untreated control group [23, 24]. For lethal time 50% (LT_50_) value calculation, data was normalized to group and non-linear regression was performed.

### Intestinal barrier analysis in *C. elegans*

Gut integrity analysis was performed by a modified Smurf staining assay using Brilliant blue FCF (C.I. 42090, Carl-Roth, Karlsruhe, Germany) as described [25]. Uniform and stable dispersion of the testing substances in NGM agar plates was ensured by applying an agar dilution method. Therefore, a defined volume of FPM was mixed with molten NGM agar in tubes. Subsequently, the tubes were thoroughly vortexed and kept at 55 °C until dispersion into 6 or 10 cm plates.

Sterile *glp-4* (SS104) worms were age-synchronized, and eggs were transferred to NGM plates and grown at 25 °C until adulthood. Next, A0 worms were separated onto either control or treatment NGM plates containing the respective amount of FPM. Plates were exchanged every 2–3 d and worms were incubated to a total age of A10. After the incubation time, freshly starved worms were washed off and incubated for 3 h without shaking at 20 °C in 1.5-mL reaction tubes containing 5% [w/v] Brilliant blue FCF in S-medium [22]. After the incubation time, worms were washed in ice-cold 1×DPBST (DPBS with 0.01% Triton X-100, Sigma-Aldrich) several times until the solution turned completely transparent. Worms were anesthetized using a 1 mmol/L levamisole hydrochloride solution (Abcam, Cambridge, UK) to avoid nematode motion. Finally, worms were pipetted onto glass slides and imaged using the Olympus SZX16 stereomicroscope and a 1× air objective (Olympus, Shinjuku, Japan). The number of Smurfs was count in each group and was represented as a fraction of the total amount of worms per group.

### *Drosophila melanogaster* husbandry

*D. melanogaster*, wild-type mutant strain *w*^*1118*^ (University of Kiel, Kiel, Germany; strain No. 5905 Bloomington *Drosophila* Stock Center, Bloomington, IN, USA), were kept in standard housing conditions (25 °C, 60% relative humidity, 12:12 light-dark cycle) on the modified CalTech medium and sugar-yeast medium previously described [26, 27].

### Detection of ROS in *D. melanogaster*

To induce oxidative stress, 25 5-day-old *w*^*1118*^ females per vial were transferred on the experimental medium containing 10% inactive dry yeast (Genesee Scientific, San Diego, CA, USA), 1.5% agar, 5% sucrose, 1% methyl 4-hydroxybenzoate solution, 0.48% propionic acid (all Carl Roth, Karlsruhe, Germany) with 30 mmol/L iron(II) sulfate (FeSO_4_) supplemented with 0.1%, 0.25%, 0.5%, 0.75%, or 1% FPM for treatment groups [27]. Control group received sugar-yeast medium without iron sulphate or FPM. Total number of the fruit flies per treatment was 225 (total of 3 experiments). After 70 h on the media, the fruit flies were sacrificed for the assay. Then, 20 µL of proteinase inhibitor cocktail (PIC, Sigma-Aldrich) was mixed with 20 mL DPBS and placed on ice. Subsequently, 300 µL of DPBS-PIC solution was added into Precellys 2-mL homogenizer tubes with 2.8 mm steel beads (Bertin Technologies SAS, Montigny-le-Bretonneux, France). The fruit flies were transferred into the homogenizer tubes and homogenized with the Precellys Evolution homogenizer (Bertin Technologies SAS) at 6,800 r/min for 40 s and placed on ice. Next, 700 µL of cold DPBS-PIC buffer was pipetted into the vial and from that, 800 µL were transferred into a 1.5-mL reaction tube. For 10 min the tubes were centrifuged at 17,000 × *g* at 4 °C and supernatants were transferred to fresh tubes and stored on ice until the assays.

To quantify the intracellular ROS level, the fluorescent probe H_2_DCFDA was used. For this, 100 µL of sample or DPBS as a blank were added to the black multiwell plate, followed by the addition of 100 µL of 10 µmol/L H_2_DCFDA solution. All the samples were assayed in triplicates. After 2 h the fluorescence intensity was measured using the POLARstar Omega microplate reader in fluorescence mode (485 nm excitation, 520 nm emission).

To normalize the obtained results from ROS assay to proteins, a Bradford assay was performed. Hence, the prepared samples were diluted 1:10 in 20 mmol/L sodium chloride solution. Finally, 40 µL of bovine serum albumin standards or samples and 160 µL of 1× Bradford reagent (Bio-Rad Laboratories) were mixed in the transparent 96-well plate in duplicates. After an incubation time of 30 min, the absorbance was measured using the POLARstar Omega microplate reader at wavelengths 595 and 450 nm.

### Intestinal barrier challenge in *D. melanogaster*

Intestinal barrier challenge was performed as previously described [27]. Twenty five 5-day-old *w*^*1118*^ females were sorted and transferred to the vials with 2% agar medium for humidity. On the cellulose acetate plugs (Genesee Scientific), a paperclip was inserted containing a piece of 1.5 mm thick blotting paper (Whatman, Maidstone, UK), which was soaked with experimental media. The fruit flies were kept in standard climate conditions for 7 d. The liquid experimental media contained 5% sucrose, 1% Brilliant Blue FCF, 3% dextran sulphate 40 sodium salt (DSS) with an average MW of 40,000 g/mol (all Carl Roth) and optionally 0.1%, 0.25%, 0.5%, 0.75%, or 1% FPM in deionized water for treatment groups. Besides the treatment groups, a control group containing only 5% sucrose and 1% Brilliant Blue FCF and a group with 3% DSS but no FPM were tested. Over a span of one week, the blotting paper soaked with liquid medium and agar vials were exchanged for fresh vials and fresh liquid medium on a new piece of blotting paper four times per experiment. The Smurf phenotype was defined as a blue coloration outside of abdominal area, suggesting blue dye “leaked” through the intestinal system. Alive and dead Smurfs, dead non-Smurfs, as well as censored fruit flies in the old and new agar vials were recorded daily starting on d 3. Total number of the fruit flies per treatment was 250 (total of 3 experiments).

### Analytical analysis

GC-MS headspace analysis was performed using a Trace 1300 gas chromatograph couples to an ISQ QD single-quadrupole mass spectrometer equipped with a PTV injector and a TriPlusRSH headspace autosampler (all Thermo Scientific, Waltham, MA, USA). 3 g of the samples were transferred to 15-mL headspace vials, pre-incubated at 70 °C for 20 min and extracted for 20 min under agitation at the same temperature using a SPME fibre assembly (30 μm CAR/PDMS, 50 µm DVB-layer, 23 ga, Supelco, St. Louis, MO, USA). Injector temperature was kept constant at 240 °C and analytes were desorbed and injected in splitless mode onto a Stabilwax-DA column (30 m × 0.25 mm, 0.25 μm film thickness; Restek, Centre County, PA, USA). Helium was used as carrier gas at a constant flow rate of 1.0 mL/min. Oven temperature was kept constant at 35 °C for 11 min, then raised to 140 °C at 10 °C/min and further to 240 °C at 25 °C/min, followed by a constant period of 5 min at 240 °C before returning to start conditions. The injector and ion source temperatures were set to 240 °C and 220 °C, respectively. Full scans from* m/z* 35 to 300 were recorded with a rate of 6.5 scans/s. Instrument operation and data analysis were performed using the Chromeleon 7.2 software package (Thermo Scientific) and analytes were tentatively identified using the NIST 11 spectral library in conjunction with the NIST 11 GC RI database [28, 29].

LC-MS analysis of the FPM was carried out using a Surveyor HPLC coupled to a LTQ Orbitrap Velos mass spectrometer (both Thermo Fisher Scientific) as described previously [30]. In brief, chromatographic separation was achieved using an Accucore C18 column (150 mm × 3 mm, 2.6 µm; Thermo Fisher Scientific) with 0.1% formic acid in water (A) and 0.1% formic acid in acetonitrile (B) as solvents. The flow rate was set to 0.5 mL/min and the following gradient was applied: 5% B for 2 min, then raise to 20% B within 6 min, 40% B within 4 min, 60% B within 3 min and 80% B within two min, followed by a hold time for 3 min, a return to 5% B within 2 min and finally 8 min of equilibration time. The mass spectrometer was equipped with an APCI ion source operated in positive mode. The resolution was set to 60,000 with a scan range from *m/z* 80 to 1,000. The instrument was operated in top 3 data dependent acquisition mode with a dynamic exclusion time of 3 min. The normalized collision-induced dissociation energy was set to 35 and acquisition of the MS/MS scans was acquired in the ion trap. Molecular formula of the compounds was calculated based on the precursor ion mass recorded with high resolution and tentative compound identification was conducted based on molecular weight, fragment ion spectra and UV absorption characteristics upon comparison to public databases [31–33].

### Statistical analysis

All statistical analyses were performed with GraphPad Prism version 9.0.0 (GraphPad Software Inc., San Diego, CA, USA). Differences between more than two groups were examined using one-way analysis of variance (ANOVA) with Dunnett’s multiple comparison test. For comparison of two groups, an unpaired *t*-test with Welch’s correction was applied. Differences were considered significant at ****P < 0.0001, ***P < 0.001, **P < 0.01, *P < 0.05 and not significant (ns) at *P* ≥ 0.05. Charts were prepared using GraphPad Prism version 9.0.0, and Corel Draw version 2019 (Corel Corporation, Ottawa, ON, Canada) was used to merge the parts of each figure. All box-and-whisker plots indicate the median, the replicates as single dots and the min/max as error bars. All bar charts and the time course of TEER values for in vitro intestinal barrier analysis indicate the mean and the SD as error bars. The time course of activity counts in *C. elegans* indicates the mean and the 95% confidence interval (CI) as area fill.

## Results

### FPM improved parameters of intestinal function in vitro

**Fig. 1:**
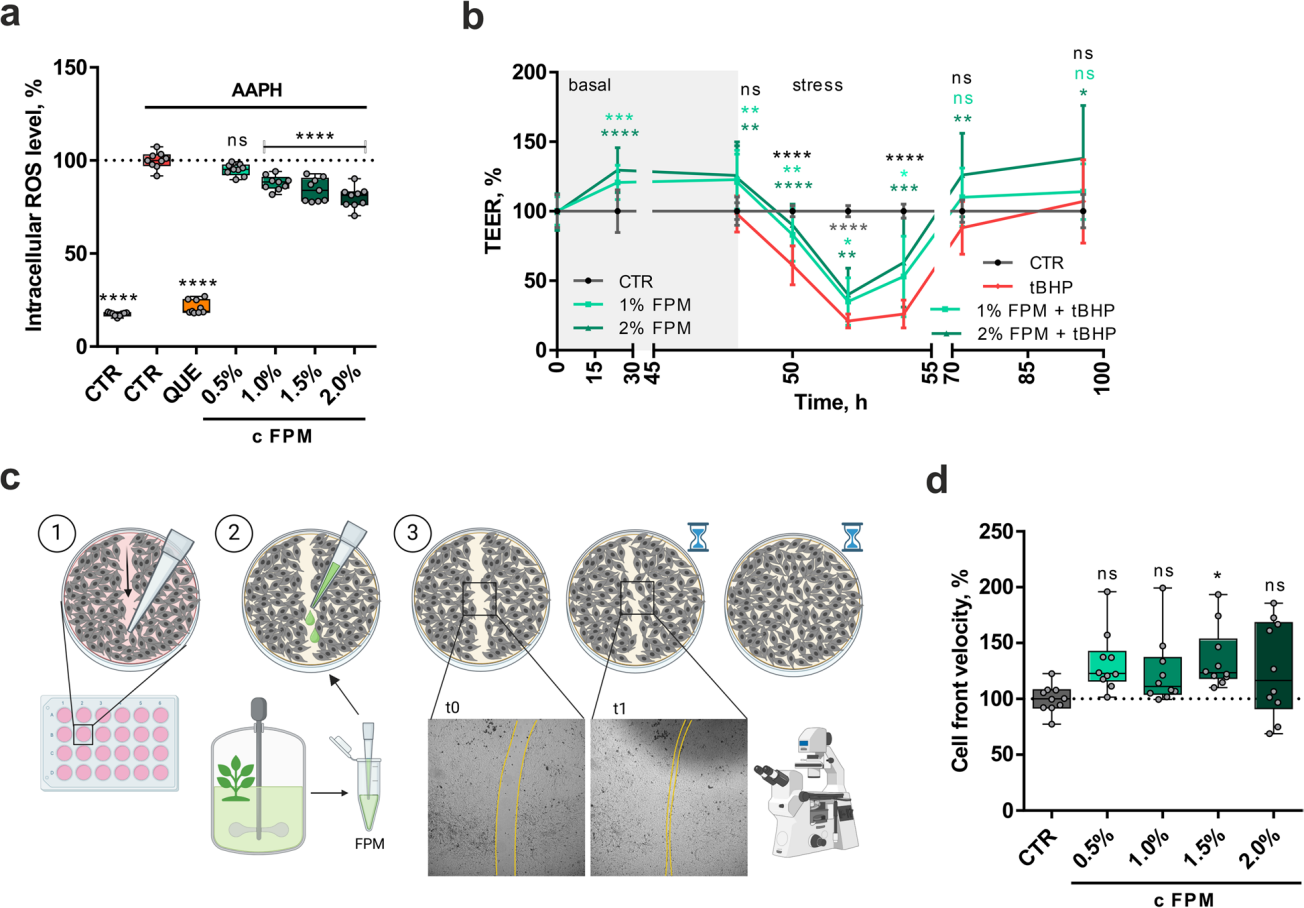
FPM reduced ROS production, strengthened barrier integrity and enhanced cell migration in IPEC-J2 cells. (**a**) ROS assay with QUE or FPM treatment and oxidative stress using AAPH. AUC of fluorescence in the stress phase represents ROS levels. Three independent experiments in triplicates (*n* = 9). (**b**) TEER values of cells on cell culture inserts recorded during FPM pretreatment and oxidative stress with tBHP. Four independent experiments in triplicates (basal phase: *n* = 24 for control and *n* = 12 for FPM; stress phase: *n* = 12). (**c**) (1) For cell migration analysis a cell monolayer in 24-well plates was scratched with a pipette tip. (2) FPM was added to the cells and (3) brightfield images were taken every 15 min. (**d**) Cell front velocity of the scratch closure. Five independent experiments in duplicates (*n* = 10). ****P < 0.0001, ***P < 0.001, **P < 0.01, *P < 0.05 and ns *P* ≥ 0.05, compared to oxidative stress control [(**a**), (**b**) stress phase] or untreated control [(**b**) basal phase, (**d**)] (one-way ANOVA).

In this study, we first evaluated the ability of FPM to improve intestinal function parameters in intestinal porcine enterocytes. Figure 1a demonstrates the percentage of intracellular ROS molecules following treatment with FPM and subsequent oxidative stress induction using the free-radical-generating compound AAPH. Additionally, QUE was included as an antioxidant positive control. FPM exhibited antioxidant effects by significantly lowering ROS generation in IPEC-J2 cells in a dose-dependent manner compared to the stress control. ROS levels were reduced by 20.01% at the highest tested concentration of 2% FPM (*P* < 0.0001).

For the analysis of barrier integrity and permeability, we documented the TEER values of a differentiated IPEC-J2 cell layer under both basal and oxidative stress conditions induced by tBHP over a 96-h period. Figure 1b illustrates the normalized TEER values of cell layers treated with 1% or 2% FPM before and after stress induction. Under basal conditions, both FPM concentrations significantly increased TEER values compared to the unstressed control for up to 48 h, indicating enhanced barrier function and decreased permeability. Upon induction of oxidative stress, TEER values declined significantly; however, this decrease was notably less pronounced in FPM-treated cells compared to the stress control. After approximately 70 h of incubation, the TEER values of the stressed cell layers began to stabilize and subsequently increased to levels comparable to the unstressed control. FPM mitigated the decrease in TEER in response to tBHP in a dose-dependent manner until the end of the 96-h measurement. Throughout the entire observation period, treatment with FPM at 2% resulted in significantly higher TEER values, suggesting that FPM effectively improves intestinal barrier integrity in vitro.

Given that oxidative stress and elevated ROS levels are linked to impaired wound healing [34], we investigated whether FPM could enhance cell migration and promote wound healing. For this, we induced mechanical stress by scratching a confluent IPEC-J2 cell monolayer with a pipette tip. The impaired cell layer was then treated with FPM, and cell migration was monitored every 15 min using automated brightfield microscopy (see Fig. 1c). To compare cell migration across treatments, we calculated the cell front velocity, defined as the speed at which cells move towards each other. As shown in Fig. 1d, the normalized cell front velocity increased with FPM treatment at the specified concentrations compared to the untreated control, although only significant at 1.5% FPM (+ 35.55%, *P* = 0.0351) due to variability between replicates. These findings indicate that FPM demonstrates significant antioxidant activity in vitro, as evidenced by a reduction in intercellular ROS production, an increase in epithelial barrier integrity and improved cell migration efficiency in porcine IPEC-J2 cells.

### FPM reduced pro-inflammatory signaling in LPS-stimulated macrophages

**Fig. 2:**
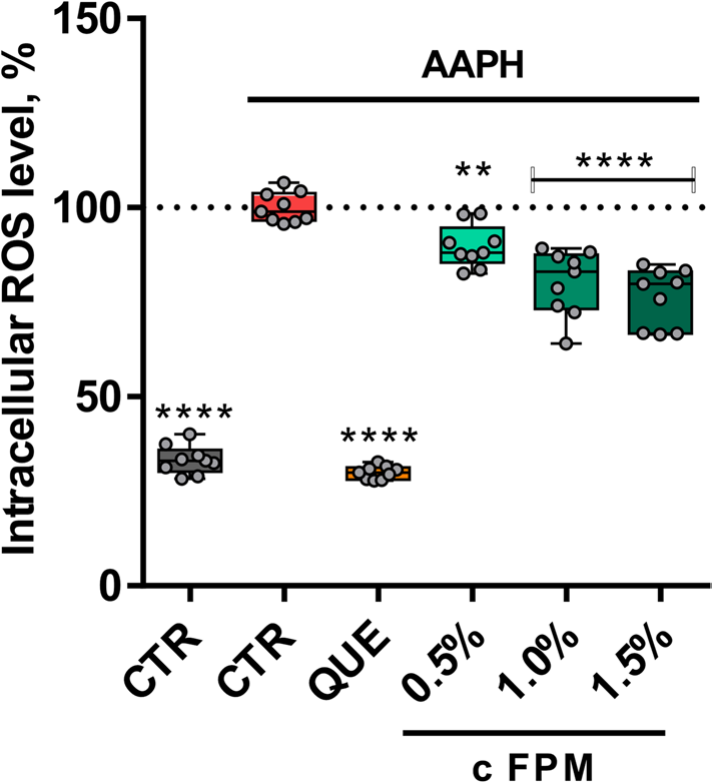
FPM reduced ROS production in THP-1 macrophages. ROS assay with QUE or FPM treatment and oxidative stress using AAPH. AUC of fluorescence in the stress phase represents ROS levels. Three independent experiments in triplicates (*n* = 9). ****P < 0.0001, **P < 0.01, compared to stress control (one-way ANOVA).

We further examined the effects of FPM on THP-1 macrophages under oxidative and inflammatory stress conditions. Consistent with its impact on ROS levels in IPEC-J2 cells, FPM significantly reduced ROS production in macrophages subjected to oxidative stress induced by AAPH, as shown in Fig. 2. Treatment with 0.5%, 1%, and 1.5% FPM mitigated the elevated ROS levels in a dose-dependent manner. The reduction at the highest tested concentration of 1.5% FPM amounted to 23.69% (*P* < 0.0001).

**Fig. 3:**
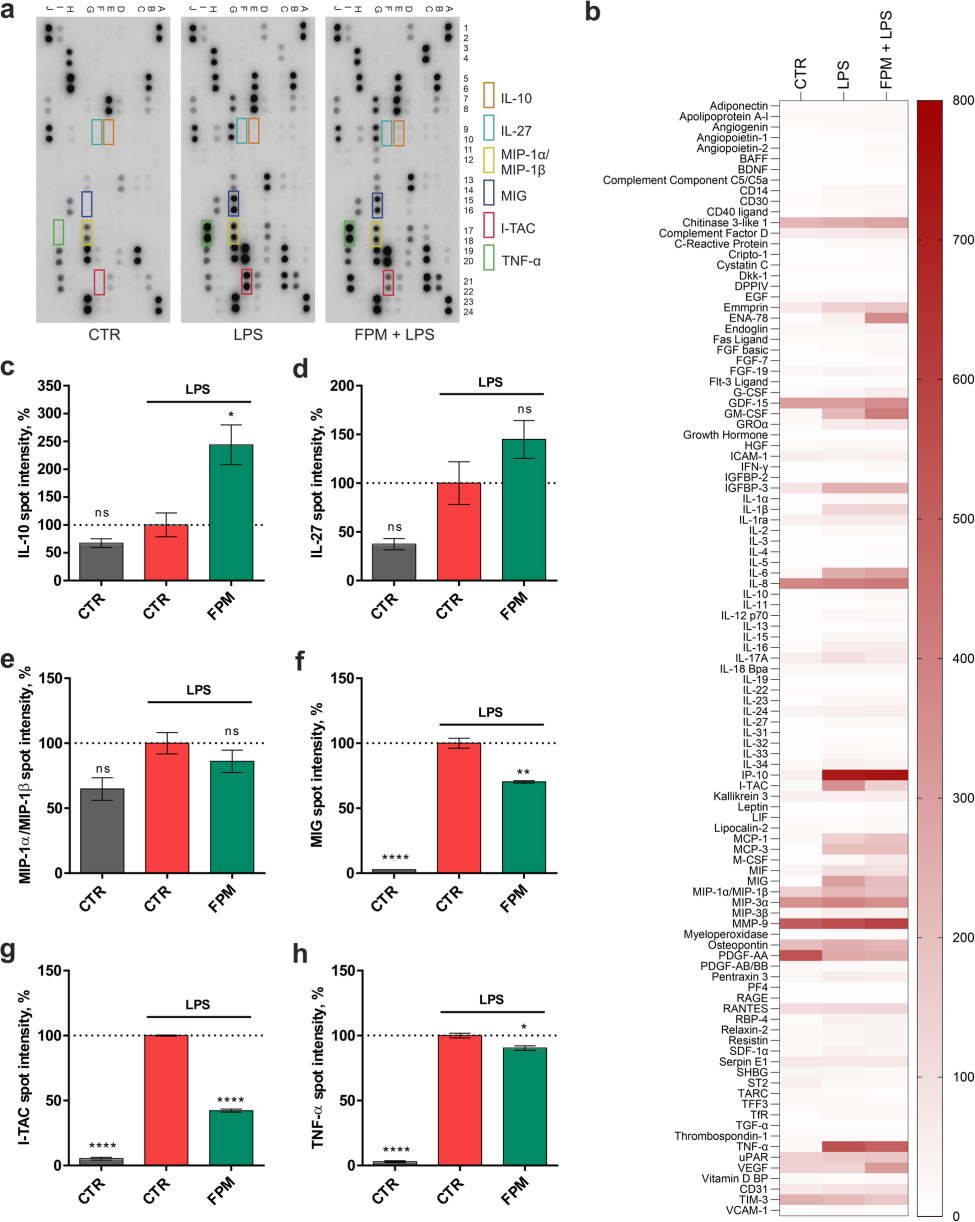
FPM modified cytokine secretion in THP-1 cells. (**a**) Membranes of a cytokine antibody array with highlighted targets selected for the multiplex assay. (**b**) Heatmap of the spot intensities of all cytokines in the antibody array. (**c**–**h**) Spot intensities of the selected targets for the multiplex assay. Duplicate spots (*n* = 2). ****P < 0.0001, **P < 0.01, *P < 0.05 and ns *P* ≥ 0.05, compared to stress control (one-way ANOVA).

To investigate the influence of FPM on inflammatory signaling, we first conducted a semi-quantitative cytokine array analysis. THP-1 macrophages were subjected to LPS to induce an inflammatory response and simultaneously treated with FPM. Membranes pre-loaded with antibodies specific to over 100 cytokines and chemokines were incubated with the supernatants from the treated THP-1 cells. The captured analytes were visualized and semi-quantified based on spot intensities. Figure 3a displays representative images of the cytokine array membranes, highlighting the analyte spots. Key analytes upregulated upon LPS challenge, compared to control treatment, are framed and numbered. The overall expression profiles of these analytes are summarized in a heat map in Fig. 3b, with corresponding spot intensity values for selected analytes presented in Fig. 3c–h. LPS stimulation resulted in the upregulation of pro-inflammatory mediators such as macrophage inflammatory protein (MIP)-1α/MIP-1β, MIG, I-TAC, and TNF-α. FPM at 1% significantly downregulated the expression of MIG (*P* = 0.002), I-TAC (*P* < 0.0001), and TNF-α (*P* = 0.0143), but had no significant effect on the elevated MIP-1α/MIP-1β expression (*P* = 0.3568) compared to the LPS control. Notably, FPM treatment also significantly increased the levels of the anti-inflammatory cytokine IL-10 (*P* = 0.0197) and by trend of IL-27 (*P* = 0.1542), which has both anti- and pro-inflammatory roles in immunity [35].

**Fig. 4:**
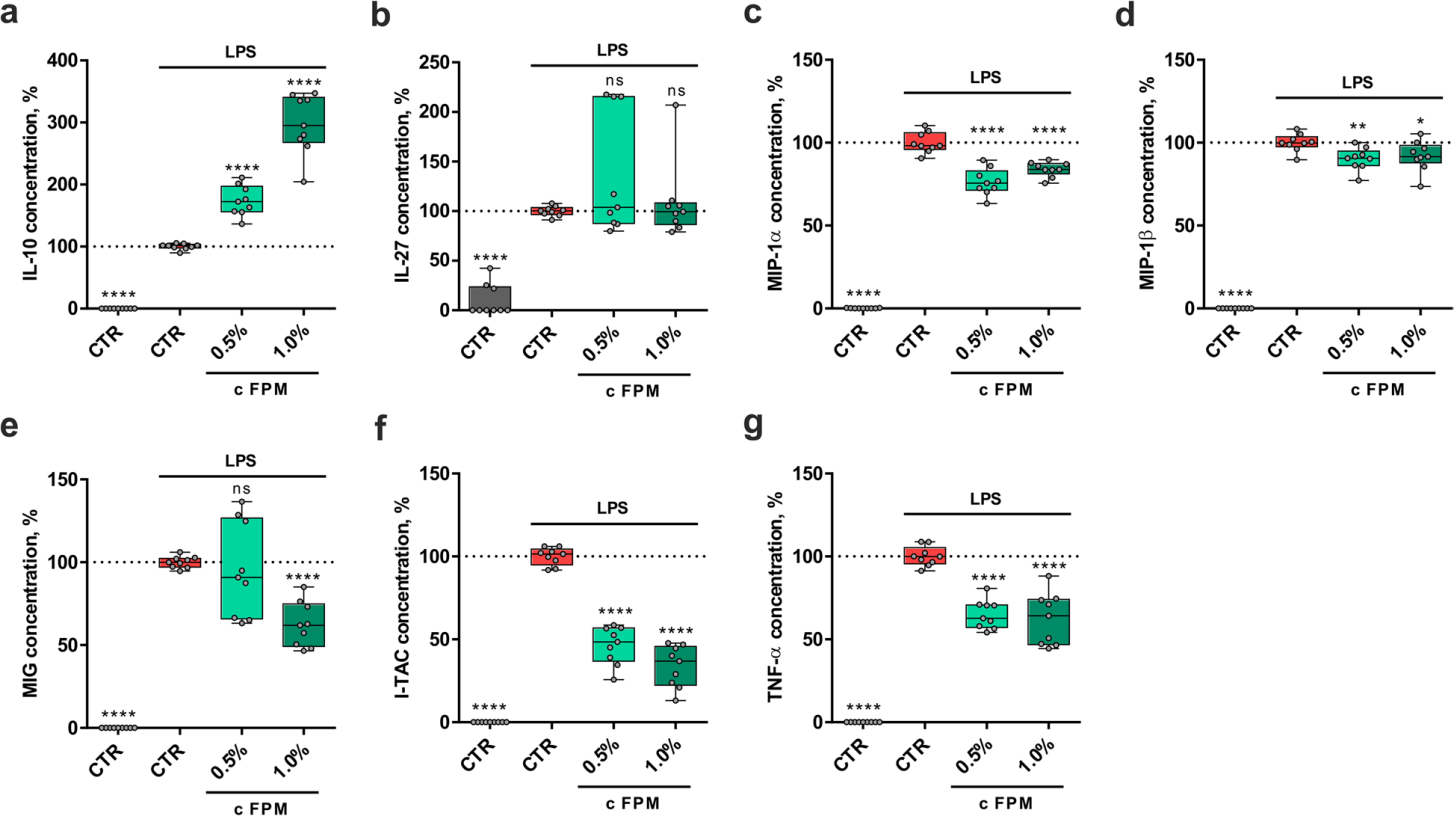
FPM altered the secretion of pro- and anti-inflammatory cytokines in LPS activated THP-1 cells. Concentrationss of the cytokines IL-10 (**a**) IL-27 (**b**), MIP-1α (**c**), MIP-1β (**d**), MIG (**e**), I-TAC (**f**) and TNF-α (**g**) in the cell supernatant after LPS and FPM treatment detected by a multiplex assay. Three independent experiments in triplicates (*n* = 9). ****P < 0.0001, **P < 0.01, *P < 0.05 and ns *P* ≥ 0.05, compared to stress control (one-way ANOVA).

To further analyze and quantify the concentrations of IL-10, IL-27, MIP-1α, MIP-1β, MIG, I-TAC, and TNF-α in the supernatant of treated THP-1 macrophages, we employed a bead-based multiplex immunoassay (see Fig. 4a–g). Consistent with the semi-quantitative results from the cytokine array analysis, 1% FPM treatment led to a significant decrease in the concentrations of MIP-1α (*P* < 0.0001), MIP-1β (*P* = 0.0268), MIG (*P* < 0.0001), I-TAC (*P* < 0.0001), and TNF-α (*P* < 0.0001), while significantly increasing the concentration of IL-10 (*P* < 0.0001). The concentrations of IL-27 varied widely between replicates of the FPM treatments thus the effect of FPM on IL-27 concentration was not significant (*P* = 0.2908).

In summary, these data suggest that FPM exerts anti-inflammatory properties by reducing the production of pro-inflammatory and prooxidant signaling molecules and enhancing the expression of specific anti-inflammatory cytokines in THP-1 macrophages.

**Fig. 5:**
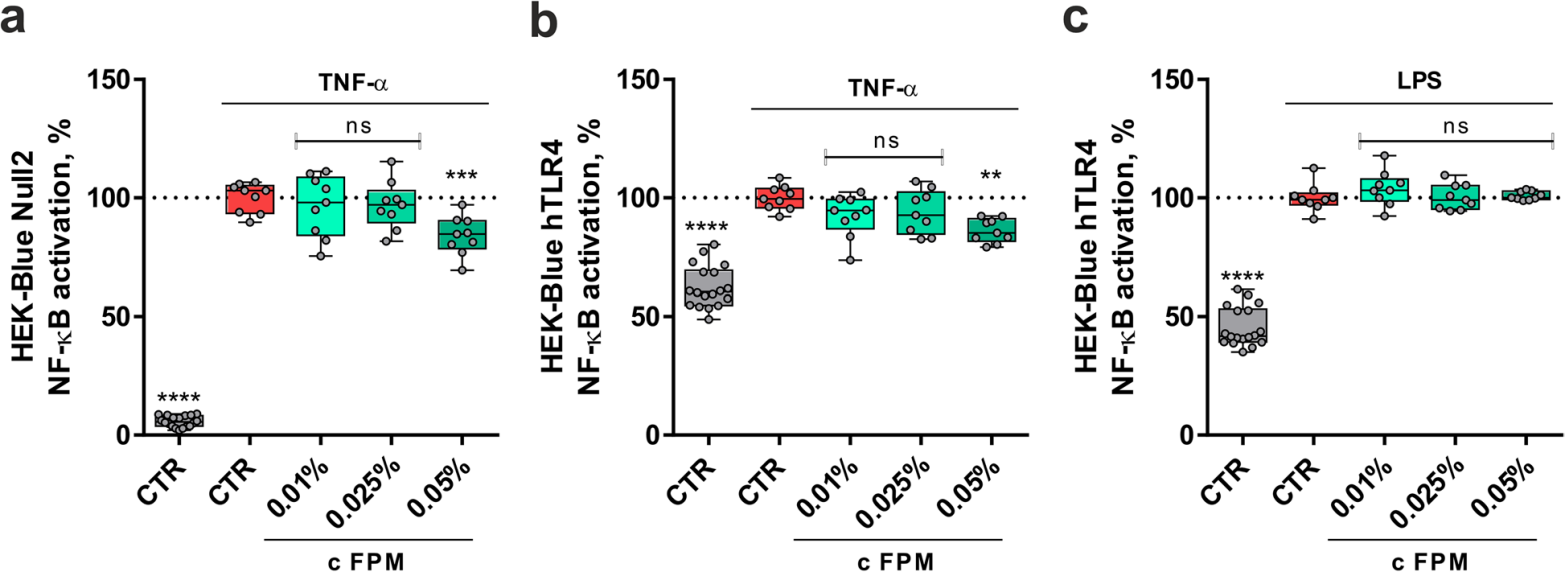
FPM triggered the NF-κB-pathway for inflammation response in HEK-Blue cells. (**a**) NF-κB activation in HEK-Blue Null2 cells with FPM treatment and TNF-α stress. (**b**) NF-κB activation in HEK-Blue hTLR4 cells with FPM treatment and TNF-α stress. (**c**) NF-κB activation in HEK-Blue hTLR4 cells with FPM treatment and LPS stress. Three independent experiments in triplicates (*n* = 9). ****P < 0.0001, ***P < 0.001, **P < 0.01 and ns *P* ≥ 0.05, compared to stress control (one-way ANOVA).

To elucidate whether the anti-inflammatory effects of FPM were mediated through the central transcription factor NF-κB via the LPS-activated TLR4 or through a TLR4-independent pathway, we utilized the TLR4 reporter cell line HEK-Blue hTLR4, which stably co-expresses the human *TLR4* gene and a SEAP reporter gene, along with its parental cell line HEK-Blue Null2, which only expresses SEAP. The production and secretion of active SEAP directly correlate with the activation of NF-κB. Figure 5a and b illustrate the NF-κB activation levels measured in the supernatant of HEK-Blue Null2 and HEK-Blue hTLR4 cells, respectively, following treatment with the stressor TNF-α. Treatment with 0.05% FPM significantly decreased elevated NF-κB activation levels in HEK-Blue Null2 and hTLR4 cells by 16.06% (*P* = 0.0003) and 13.88% (*P* = 0.0019), respectively, compared to the TNF-α control. Conversely, FPM did not decrease NF-κB activation following stress induction with LPS (see Fig. 5c), indicating that FPM reduces NF-κB activation and signaling in a TLR4-independent manner.

### FPM reduced oxidative stress but did not impact intestinal barrier integrity in *C. elegans *and* D. melanogaster*

**Fig. 6:**
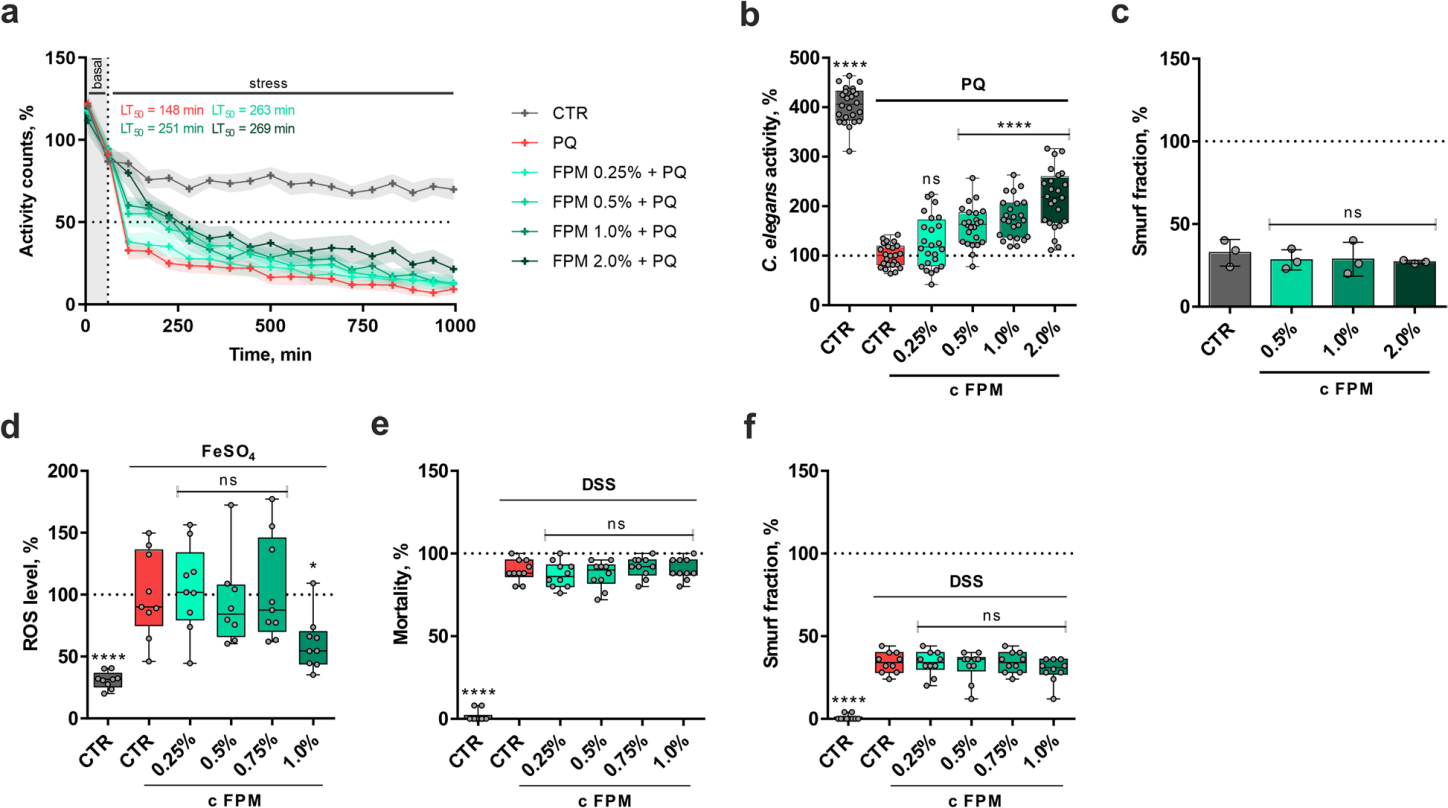
FPM reduced oxidative stress but did not improve intestinal barrier integrity in *C.* *elegans* and *D. melanogaster*. (**a**) *C. elegans* activity assay with FPM treatment and oxidative stress using PQ. Three independent experiments with eight wells (*n* = 24). (**b**) Activity counts over time were summarized with the AUC to represent the antioxidant capacity. Three independent experiments with eight wells (*n* = 24). (**c**) Smurf assay for intestinal barrier investigation in *C.* *elegans* with FPM treatment. Three independent experiments (*n* = 3). (**d**) ROS assay in *D. melanogaster* with FPM treatment and oxidative stress using FeSO_4_. Fluorescence represents ROS levels. Three independent experiments in triplicates (*n* = 9). (**e**) Mortality analysis in *D. melanogaster* with FPM treatment and intestinal stress using DSS. Three independent experiments with 3-4 replicates (*n* = 10). (**f**) Smurf assay for intestinal barrier investigation in *D. melanogaster* with FPM treatment and intestinal stress using DSS. Three independent experiments with 3–4 replicates (*n* = 10). ****P < 0.0001, *P < 0.05 and ns *P* ≥ 0.05, compared to oxidative stress control (**b**, **d**, **e**, **f**) or untreated control (**c**) (one-way ANOVA)

Based on the in vitro results, we extended our investigation to evaluate the antioxidant and intestinal barrier protective properties of FPM in the in vivo model organisms *C. elegans* and *D. melanogaster*. The nematode *C. elegans* shares many conserved biological pathways with higher organisms, allowing for the extrapolation of findings to more complex systems and is therefore widely accepted for studying molecular mechanisms in eukaryotic organisms [36]. We analyzed the protective effects of FPM on *C. elegans* under oxidative stress induced by PQ. Locomotive motility over time served as the indicative readout parameter. Figure 6a presents the activity counts of untreated worms, stressed worms, and stressed worms treated with FPM over time. PQ-induced oxidative stress significantly decreased worm activity, which was counteracted by FPM. Worms pretreated with 0.5%, 1% and 2% FPM tolerated PQ-induced oxidative stress significantly longer than the PQ control group. Non-linear regression analysis revealed an increase in LT_50_-values, ranging from 251 to 269 min, compared to the PQ control (LT_50_ = 148 min), suggesting improved lifespan of *C. elegans* under oxidative stress. The respective area under the curve (AUC) values of the individual time courses are summarized in Fig. 6b. A significant and dose-dependent increase in worm activity under oxidative stress was documented in the worms treated with 0.5%, 1% and 2% FPM, relative to the stress control. At the highest tested concentration of 2% FPM, the activity of *C. elegans* was increased by 115% (*P* < 0.0001).

To assess intestinal barrier integrity in *C. elegans*, we conducted a Smurf assay using Brilliant Blue FCF dye. Smurfs serve as models for leaky gut syndrome, as the non-absorbable dye penetrates surrounding tissues after ingestion in leaky guts, thereby staining the entire organism blue. We investigated whether FPM could modulate age-related disruption of gut integrity in *C. elegans*. The number of Smurfs was counted in each treatment group and expressed as a fraction of the total worm population per group. As depicted in Fig. 6c, treatment with 0.5%, 1%, or 2% FPM did not significantly enhance intestinal barrier integrity. These results suggest that while FPM effectively mitigates PQ-induced oxidative stress in *C. elegans*, it does not affect intestinal barrier integrity under the experimental conditions of our study.

To investigate these effects in a different in vivo model, we utilized *D. melanogaster* to study oxidative and intestinal stress. *D. melanogaster* is a robust model for examining oxidative stress and intestinal health, often preferred over mammalian models due to its genetic relevance and simplicity of cultivation. The intestinal structure and functions of *D. melanogaster* share numerous similarities with mammalian systems, making it an ideal model for such studies [37]. To evaluate the antioxidant effects of FPM, we induced oxidative stress in female *D. melanogaster* by supplementing their diet with FeSO_4_. ROS formation was quantified after 70 h of induced oxidative stress. As shown in Fig. 6d, treatment with 1% FPM significantly reduced ROS levels in *D. melanogaster* by 39.23% (*P* = 0.0471), compared to the stress-only group. This finding is consistent with the results observed in the *C. elegans* oxidative stress model, further confirming the antioxidant activity of FPM in vivo.

The Smurf assay was also applied in *D. melanogaster* and leaky gut syndrome was induced using DSS, a compound known to compromise intestinal barrier integrity in the fruit flies [38]. The number of Smurf flies and mortality rates were recorded over a 7-day treatment period. Compared to the DSS stress control, treatment with FPM did not result in a significant decrease in mortality or Smurf phenotypes (see Fig. 6e and f), indicating no improvement of gut integrity. These findings align with the results of the intestinal barrier integrity analysis in *C. elegans*.

In summary, while FPM demonstrated antioxidant activity in the in vivo models *C. elegans* and *D. melanogaster*, it did not affect intestinal barrier integrity in these models.

### Analysis of active components

**Fig. 7:**
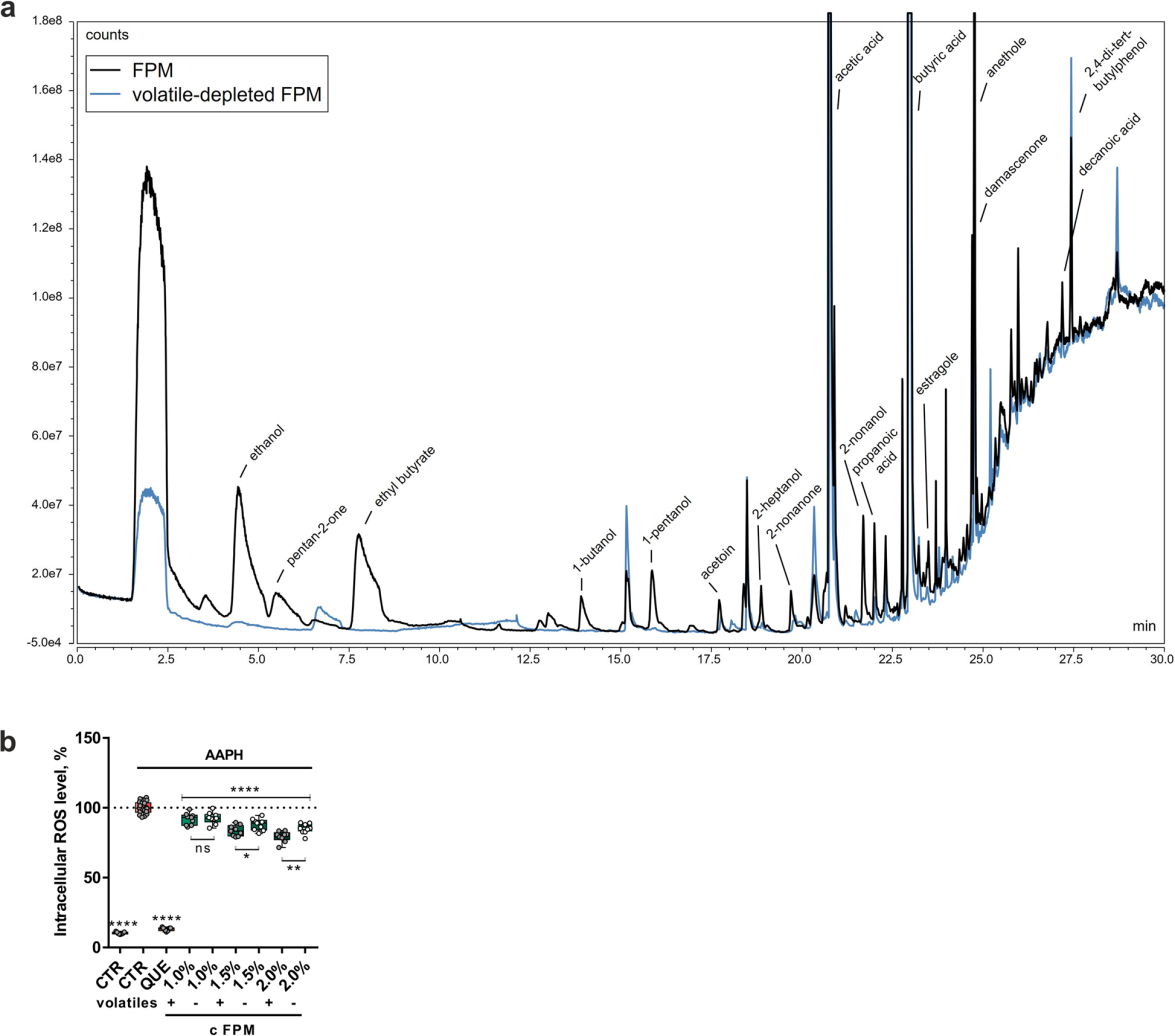
GC-MS and ROS inhibition analysis of FPM with and without volatile compounds*.* (**a**) GC-MS chromatograms comparing FPM and volatile-depleted FPM. (**b**) ROS inhibition assay in IPEC-J2 cells comparing antioxidant activities of FPM and FPM without volatile compounds. Three independent experiments in triplicates (*n* = 9). ****P < 0.0001, compared to oxidative stress control (one-way ANOVA). For comparison of FPM with and without volatiles **P < 0.01, *P < 0.05 and ns *P* ≥ 0.05 (unpaired *t*-test)

To identify active components in FPM, we performed HPLC-MS analysis, which revealed key compounds, including 4-hydroxybenzoic acid, sinapaldehyde, syringic acid, and apigenin-O-hexosyl-O-pentoside (see Additional file 1, Fig. S1 and Table S2). To further identify volatile compounds, we used GC-MS on FPM and compared it to a vacuum-concentrated, volatile-depleted FPM control. Fig. 7a shows the GC-MS chromatograms, highlighting a substantial reduction in key volatile compounds in the treated sample, including ethanol, pentan-2-one, ethyl butyrate, 1-butanol, 2-heptanol, 2-nonanol, propanoic acid, butyric acid, damascenone, anethole, and decanoic acid (see Table 1). HPLC-MS analysis of the volatile-depleted FPM showed no differences in the non-volatile compound profile compared to untreated FPM, indicating that the pre-treatment selectively reduced volatiles without affecting other constituents (see Additional file 1, Fig. S1 and Table S2).

**Table 1:** Comparison of volatile compound presence in FPM and FPM without volatiles

Compound	Retention time, min	Area, counts × min	
		FPM	FPM without volatiles
Ethanol	4.435	1.69 × 10^7^	8.90 × 10^5^
Pentan-2-one	5.475	3.78 × 10^6^	n.a.
Ethyl butyrate	7.766	1.47 × 10^7^	n.a.
1-Butanol	13.903	1.52 × 10^6^	n.a.
Acetoin	17.718	8.41 × 10^5^	2.31 × 10^5^
2-Heptanol	18.394	1.02 × 10^6^	n.a.
2-Nonanone	18.394	9.30 × 10^5^	n.a.
Acetic acid	20.746	3.58 × 10^7^	1.13 × 10^7^
2-Nonanol	21.691	1.87 × 10^6^	n.a.
Propanoic acid	21.996	1.51 × 10^6^	3.93 × 10^5^
Butyric acid	22.944	2.51 × 10^8^	7.74 × 10^7^
Estragole	23.488	8.41 × 10^5^	2.17 × 10^5^
Damascenone	24.695	3.05 × 10^6^	n.a.
Anethole	24.759	9.42 × 10^6^	3.50 × 10^6^
Decanoic acid	27.186	7.40 × 10^5^	4.29 × 10^5^
2,4-di-tert-butylphenol	27.427	2.21 × 10^6^	2.83 × 10^6^

"n.a." indicates compounds not detected in FPM without volatiles

To determine if these volatile compounds contribute to FPM’s bioactivity, we assessed the antioxidant activity of both FPM and volatile-depleted FPM in IPEC-J2 cells by measuring ROS inhibition. As shown in Fig. 7b, FPM at 1.5% (*P* = 0.0355) and 2% (*P* = 0.0054) exhibited significantly greater ROS inhibition compared to FPM without volatiles, suggesting that the volatile compounds play an important role in FPM’s antioxidant activity. However, FPM without volatiles significantly decreased ROS production as well, indicating that its non-volatile compounds and potentially other unidentified constituents contribute to its antioxidant effects. Given the complex nature of FPM’s composition, synergistic effects among these compounds are highly plausible.

## Discussion

Maintaining gut health in livestock is crucial for optimizing nutrient absorption, enhancing growth performance, and reducing susceptibility to diseases, ultimately improving overall productivity and welfare [39]. Oxidative stress and inflammation can damage the gut lining, leading to impaired nutrient uptake and increased vulnerability to gastrointestinal disorders [40]. In this study, we aimed to analyze the impact of FPM, comprising 45 distinct local plants, on gut health by evaluating its potent antioxidant, anti-inflammatory, and intestinal barrier-strengthening properties both in vitro and in vivo. Our findings demonstrate that FPM can support intestinal health by mitigating oxidative stress, reducing pro-inflammatory signaling, and improving the integrity of intestinal cell monolayers.

First, we assessed the impact of FPM on parameters of intestinal functionality using the intestinal porcine enterocyte cell line IPEC-J2. Many herbal extracts, such as those from raspberry leaves (*Rubus idaeus*), lemon balm (*Melissa officinalis*), and rosemary (*Salvia rosmarinus*), all of which are components of FPM, are known for their ability to mitigate intracellular ROS production under oxidative stress conditions [19, 41]. Bioactive compounds from different plants often exhibit additive and synergistic effects, therefore combining several herbs could increase the variety of antioxidants and thus the antioxidant capacity of the extract [42]. Recent studies have shown that fermentation can further enhance the antioxidant potential of herbal extracts for instance by improving cellular ROS inhibition and superoxide dismutase-like activity [43]. Li et al. [44] documented a significant increase in total antioxidant capacity and levels of glutathione peroxidase, a key antioxidant enzyme, in IPEC-J2 cells treated with a fermented herbal extract containing five commonly used herbs in traditional Chinese medicine. These findings underscore the potential of FPM to mitigate oxidative stress and to enhance the overall antioxidant defense system, thereby promoting intestinal health. In previous studies, we established a link between elevated ROS levels and the subsequent impairment of intestinal barrier integrity and cell migration under stress conditions. These detrimental effects were shown to be reversible with the application of specific plant extracts [26, 45]. Consistent with our earlier findings, FPM treatment significantly reduced intracellular ROS levels and restored both barrier integrity and cell migration efficiency in IPEC-J2 cells. These findings provide evidence for the gut health-promoting effects of a fermented mixture of locally cultivated herbs, leveraging the inherent microbiota of the plants to enhance their bioactive potential.

Given the promising results from the IPEC-J2 oxidative stress model, we extended our investigation to the THP-1 inflammation model to further explore the anti-inflammatory properties of FPM. THP-1 macrophages were chosen due to their critical role in the intestinal immune response, where they act as key regulators of inflammation. Macrophages are essential for maintaining gut homeostasis, responding to microbial infections, and resolving inflammation [46]. By assessing the impact of FPM on THP-1 cells, we aimed to understand its potential in modulating macrophage-mediated inflammatory pathways, thereby providing a comprehensive view of its effects on gut health. Treatment with FPM also significantly decreased ROS levels under stress conditions in THP-1 cells. ROS are known to activate inflammatory pathways, including NF-κB, which plays a critical role in the regulation of immune responses and the production of pro-inflammatory cytokines [47]. We found that FPM effectively modulated LPS-induced inflammatory responses by significantly inhibiting the production of certain pro-inflammatory cytokines, while simultaneously increasing the production of anti-inflammatory mediators. These results align with previous studies on the anti-inflammatory and immunomodulatory properties of plant-based fermented foods [48]. For instance, a fermented *Paeoniae alba* extract with *Lactobacillus brevis* 174A suppressed the concentration of the inflammatory cytokines IL-6 and TNF-α and downregulated inducible nitric oxide synthase (*iNOS*), *IL-6*, *TNF-α*, and *IL-1* gene expressions compared to the unfermented extract [49]. Similarly, a fermented *Chenopodium formosanum* leaf extract with *Aspergillus oryzae* reduced nitric oxide, IL-6, and TNF-α production in LPS-stimulated RAW264.7 cells in a dose-dependent manner [50]. Furthermore, fermented extracts combining multiple herbs have also shown significant anti-inflammatory effects. For example, fermented Chinese herbal medicines significantly increased the relative mRNA expression of the intestinal anti-inflammatory factors transforming growth factor beta (*TGF-β*) and *IL-10*, while decreasing the pro-inflammatory factors *IL-8*, *IL-15*, and *TNF-α* in juvenile largemouth bass [51]. In our study, FPM increased IL-10 levels, consistent with these findings, highlighting its potential to enhance anti-inflammatory responses. However, there is limited information available on the specific cytokines we tested and the use of fermented plant products as feed supplements in livestock. The extraction process used in our study adds complexity and distinctiveness to FPM. The herbs were extracted through maceration, involving soaking in drinking water with added mineral salts under alkaline conditions. This was followed by fermentation using organic sugar cane molasses, which promoted the growth of the plants’ native microbiota. This approach not only led to natural acidification and stabilization of the extract but also likely preserved a broader spectrum of bioactive compounds that might be altered or lost in conventional methods. This distinctive combination of maceration and fermentation, utilizing the plants’ inherent microbiota, complicates direct comparisons with existing literature, where different extraction and fermentation techniques are typically used. Most studies either use unfermented extracts or rely on the introduction of specific strains of bacteria or fungi to drive fermentation, leading to potentially different phytochemical profiles and bioactivities.

To elucidate the mechanisms underlying the inhibitory effects of FPM on pro-inflammatory signaling, we examined its impact on TLR4 and NF-κB activation using HEK-Blue reporter cell lines. By utilizing two cell lines with one stably expressing the *TLR4* gene and one lacking *TLR4* expression, we demonstrated that FPM exerted its inhibitory effect on NF-κB activation through a TLR4-independent pathway, indicating a TNF-α-mediated mechanism instead. Using cytokine array analysis and multiplex immunoassays, we identified a significant reduction in TNF-α levels following FPM treatment, which likely plays a partial role in the observed suppression of TNF-α-induced NF-κB activation. Few herbal extracts have been shown to reduce NF-κB activation. For instance, a water extract containing eight different herbs, none of which are included in FPM, was shown to inhibit NF-κB activation and iNOS promoter activity. This extract also inhibited the secretion of NF-κB-dependent pro-inflammatory cytokines TNF-α and IL-1β in LPS-stimulated RAW264.7 cells [52]. Conversely, studies on fermented *Mercurialis perennis* extract with *Lactobacillus plantarum* and *Pediococcus pentosaceus* and its non-fermented counterpart revealed a concentration-dependent immunostimulatory effect. These extracts enhanced NF-κB and cytokine expression (IL-6, TNF-α, IL-8, and IL-1) in NF-κB-THP-1 reporter cells [53]. This contrast highlights the diverse effects of herbal extracts depending on their specific compositions and fermentation processes. Importantly, to the best of our knowledge, our study is the first to indicate a TLR4-independent mechanism of action of a fermented herbal extract.

Having established the effects of FPM on oxidative stress and inflammatory pathways in vitro, we next aimed to validate these findings in in vivo models to assess the physiological relevance of FPM’s antioxidant and intestinal barrier strengthening properties. *C. elegans* and *D. melanogaster* were chosen for this purpose due to their well-characterized responses to oxidative stress and their relevance in studying intestinal health. *C. elegans* offers a powerful model for examining the impact of oxidative stress on longevity and gut integrity, while *D. melanogaster* provides insights into antioxidant effects and intestinal function within a more complex organismal context [36, 37, 54]. We found that FPM significantly reduced ROS production in *D. melanogaster* and enhanced both worm activity and LT_50_ values in *C. elegans*, suggesting increased lifespan*,* under oxidative stress. These findings indicate strong antioxidant properties of FPM in vivo. Few plant extracts have been previously described to decrease ROS levels in *D. melanogaster*. For instance, extracts from *Rhodiola crenulata* and *Punica granatum* demonstrated antioxidant activity by effectively scavenging ROS molecules [55, 56]. Additionally, our recent research showed that fermented citrus extract significantly inhibited ROS production in *D. melanogaster*, attributed to the increased concentration of deglycosylated flavonoids resulting from fermentation by lactic acid bacteria [27]. In *C. elegans*, treatment with fermented coix seed polysaccharides was shown to enhance antioxidant enzyme activities (superoxide dismutase and catalase) and significantly increase lifespan under oxidative and heat stress [57]. Similar results were documented by Wang et al. [58], who additionally showed that orange extract could delay the decline of worm motility in a dose-dependent manner. However, the literature on the antioxidant activity of fermented extracts in *D. melanogaster* and *C. elegans* remains limited. In our study, we did not observe positive or negative effects of FPM on intestinal barrier integrity in either in vivo model under our experimental conditions. Previously, we have shown improved intestinal integrity after treatment with plant-based extracts using similar experimental setups [26, 59]. In the present study, the Smurf assay was employed in *C. elegans*, a method that has been underexplored with only a limited number of studies published to date [25, 60]. This assay was conducted without chemically induced stress, meaning that any increase in Smurf phenotypes would reflect changes associated with natural aging rather than stress-induced intestinal permeability. In contrast, our in vitro studies demonstrated that FPM protected the intestinal barrier under chemically induced stress, suggesting that the absence of a stressor in *C. elegans* may have limited our ability to detect similar protective effects. The lack of well-established chemical stressors for inducing intestinal permeability in *C. elegans* represents a current challenge. However, incorporating such stressors in future studies could provide deeper insights into FPM’s effects under stress conditions. In *D. melanogaster*, flies were treated with FPM while being simultaneously exposed to dextran sulfate sodium (DSS) through their diet. This approach differs from our in vitro experiments, where cells were pre-treated with FPM before the stressor was applied. In vitro, pre-treatment increased TEER values, and although TEER decreased upon stressor addition, it remained higher than in cells exposed to the stressor alone. These findings suggest that pre-treatment enables FPM to activate protective pathways before the onset of stress. However, in *D. melanogaster*, simultaneous treatment with FPM and DSS may not provide sufficient time for such protective mechanisms to be established. These observations underscore the importance of experimental design, particularly regarding the timing and duration of treatment, in determining FPM’s protective effects.

Future studies could focus on introducing chemical stressors in *C. elegans* to better mimic stress conditions, as well as testing pre-treatment protocols in *D. melanogaster* to assess the impact of treatment timing on intestinal barrier integrity. Zhang et al. [61] recently reported a decrease in Smurf phenotypes in *D. melanogaster* caused by aging following treatment with purple sweet potato extract. However, available literature on this subject is limited.

The analytical characterization of FPM using HPLC-MS and GC-MS provided valuable insights into its chemical composition, which may underlie its observed bioactivities. Several phenolic compounds identified through HPLC-MS, such as syringic acid and 4-hydroxybenzoic acid, have been previously reported in the literature for their potent antioxidant properties, suggesting their potential contribution to the ROS-scavenging effects of FPM [62, 63]. Moreover, the GC-MS analysis revealed the presence of volatile compounds commonly found in essential oils, such as estragole and damascenone, which are known for their antioxidant and immunoregulatory activities [64, 65]. These compounds, in synergy with other identified and unidentified constituents, could play a role in modulating oxidative stress and inflammatory pathways. The presence of bioactive volatile and non-volatile compounds highlights the complexity of FPM’s phytochemical profile and suggests that its functional properties may arise from multifaceted interactions between its components rather than the effects of individual compounds alone.

While these findings provide important insights, animal tests and feeding trials remain crucial for several reasons. First, the complexity of livestock systems, including the interactions between diet, microbiota, and host physiology, cannot be fully replicated in simpler organisms like *C. elegans* and *D. melanogaster*. Livestock species have unique digestive systems and metabolic processes that could affect the bioavailability and efficacy of FPM [66, 67]. Moreover, the impact of FPM on production parameters such as growth performance, feed efficiency, and overall animal health can only be accurately assessed in the target species. Feeding trials in livestock will also help to determine the optimal dosage, safety, and long-term effects of FPM. Therefore, while our current findings indicate that FPM shows promising potential as a functional feed supplement for livestock, particularly in enhancing gut health and resilience to oxidative and inflammatory stress, further research, including comprehensive animal trials, is essential to fully elucidate the benefits of FPM in livestock production.

## Conclusion

Our study demonstrates that FPM, composed of 45 locally sourced plants, exhibits significant antioxidant and anti-inflammatory properties in both in vitro and in vivo models by reducing ROS levels, inhibiting pro-inflammatory signaling and strengthening the epithelial barrier integrity of enterocytes under stress. Through leveraging the inherent microbiota of the plants, the fermentation process preserves the integrity and potency of the herbal components, while promoting a sustainable production process. This approach underscores the importance of utilizing local biodiversity and traditional fermentation techniques to optimize the health benefits of herbal supplements. These findings suggest that FPM holds promise as a functional feed supplement for livestock, potentially enhancing gut health and resilience to oxidative and inflammatory stress. However, further research, including feeding trials, is necessary to confirm these benefits in practical livestock settings and to fully understand the mechanisms underlying the observed effects.

## Supplementary Information

Additional file 1: Table S1. Composition of herbs used in FPM formulation. The table lists each herb included in the formulation by common and binomial name, along with the proportion (%) of each herb in the total extract composition. Fig. S1. Overlay of HPLC-DAD chromatograms of FPM and volatile-depleted FPM. Injection volume was 1 µL each and detection wavelength was set to 260 nm. Compounds were tentatively identified based on the HR-MS measurements as listed in Table S2. Table S2. Overview of the tentatively identified compounds in FPM through HPLC-MS.

## Data Availability

Data are available from the corresponding authors upon reasonable request.

## Abbreviations

AAPH: 2,2´-Azobis(2-methylpropionamidine) dihydrochloride
ANOVA: Analysis of variance
AUC: Area under the curve
BSA: Bovine serum albumin
CGC: *C. elegans* Genetics Center
DMEM: Dulbecco’s Modified Eagle Medium
DPBS: Dulbecco’s phosphate-buffered saline
DPBST: DPBS with 0.01% Triton X-100
DSS: Dextran sulphate 40 sodium salt
FBS: Fetal bovine serum
FPM: Fermented plant macerate
H_2_DCF: 2´,7´-Dichlorodihydrofluorescein
H_2_DCFDA: 2´,7´-Dichlorodihydrofluorescein diacetate
HBSS: Hank’´s balanced salt solution
hTLR4: Human Toll-like receptor 4
IL: Interleukin
iNOS: Inducible nitric oxide synthase
IPEC-J2: Intestinal porcine epithelial cells-jejunum 2
I-TAC: Interferon-inducible T cell alpha chemoattractant
LPS: Lipopolysaccharides
LT_50_: Lethal time 50%
MIG: Monokine induced by gamma interferon
MIP: Macrophage inflammatory protein
NF-κB: Nuclear factor kappa B
NGM: Nematode growth medium
PEG: Polyethylenglycol
PIC: Proteinase inhibitor cocktail
PMA: Phorbol 12-myristate 13-acetate
PQ: Paraquat
QUE: Quercetin
ROS: Reactive oxygen species
RPMI 1640: Roswell Park Memorial Institute 1640
SEAP: Secreted embryonic alkaline phosphatase
tBHP: Tert-butyl hydroperoxide
TEER: Trans-epithelial electrical resistance
TGF-β: Transforming growth factor beta
THP-1: Tohoku Hospital Pediatrics-1
TLR4: Toll-like receptor 4
TNF-α: Tumor necrosis factor alpha
