# Supplementary material for: Herbal extract fermented with inherent microbiota improves intestinal health by exerting antioxidant and anti-inflammatory effects in vitro and in vivo
Source: J Anim Sci Biotechnol. 2025 Apr 6;16:52. doi: 10.1186/s40104-025-01178-w (PMC11972464; doi:10.1186/s40104-025-01178-w)
Supplement: Supplementary file 1 — Additional file 1: Table S1. Composition of herbs used in FPM formulation. The table lists each herb included in the formulation by common and binomial name, along with the proportion (%) of each herb in the total extract composition. Fig. S1. Overlay of HPLC-DAD chromatograms of FPM and volatile-depleted FPM. Injection volume was 1 µL each and detection wavelength was set to 260 nm. Compounds were tentatively identified based on the HR-MS measurements as listed in Table S2. Table S2. Overview of the tentatively identified compounds in FPM through HPLC-MS. [file 40104_2025_1178_MOESM1_ESM.docx]

**Additional file 1**

**Table S1**: Composition of herbs used in FPM formulation. The table lists each herb included in the formulation by common and binomial name, along with the proportion (%) of each herb in the total extract composition.

| **Name** | **Binomial name** | **Ratio** |
| --- | --- | --- |
| Valerian | *Valeriana officinalis* | 3.25% |
| Apple mint | *Mentha suaveolens* | 4.00% |
| Russian comfrey | *Symphytum × uplandicum* | 3.75% |
| Common nettle | *Urtica dioica* | 4.00% |
| Blackberry leaves | *Rubus fruticosus* | 2.50% |
| Oregano | *Origanum vulgare* | 2.50% |
| Southernwood | *Artemisia abrotanum* | 1.25% |
| Lady´s mantle | *Alchemilla vulgaris* | 3.00% |
| Ground elder | *Aegopodium podagraria* | 2.50% |
| European goldenrod | *Solidago virgaurea* | 1.00% |
| Raspberry leaves | *Rubus idaeus* | 2.50% |
| Shepherd´s purse | *Capsella bursa-pastoris* | 0.75% |
| Elder leaves | *Sambucus nigra* | 4.00% |
| Dwarf nasturtium | *Tropaeolum minus* | 2.50% |
| Lavender | *Lavandula angustifolia* | 1.00% |
| Lovage | *Levisticum officinale* | 2.75% |
| Lungwort | *Pulmonaria officinalis* | 0.50% |
| Meadowsweet | *Filipendula ulmaria* | 3.00% |
| Marjoram | *Origanum majorana* | 2.50% |
| Greater musk-mallow | *Malva alcea* | 2.00% |
| Lemon balm | *Melissa officinalis* | 4.00% |
| Peppermint | *Mentha × piperita* | 4.00% |
| Vitex | *Vitex agnus-castus* | 1.00% |
| Common evening-primrose | *Oenothera biennis* | 1.50% |
| Fragrant agrimony | *Agrimonia procera* | 1.50% |
| Rhubarb | *Rheum rhabarbarum* | 4.00% |
| Common Marigold | *Calendula officinalis* | 3.75% |
| Rosemary | *Salvia rosmarinus* | 0.75% |
| Rocket | *Eruca vesicaria* | 1.25% |
| Common sage | *Salvia officinalis* | 4.00% |
| Yarrow | *Achillea millefolium* | 2.50% |
| Greater celandine | *Chelidonium majus* | 1.00% |
| Ribwort plantain | *Plantago lanceolata* | 3.75% |
| Cicely | *Myrrhis odorata* | 2.00% |
| Yellow archangel | *Lamium galeobdolon* | 1.25% |
| Lemon thyme | *Thymus citriodorus* | 1.25% |
| Common chicory | *Cichorium intybus* | 1.50% |
| Square stalked willow herb | *Epilobium tetragonum* | 0.75% |
| Rue | *Ruta graveolens* | 2.50% |
| Small burnet | *Sanguisorba minor* | 1.00% |
| Betony | *Betonica officinalis* | 0.75% |
| Hyssop | *Hyssopus officinalis* | 1.50% |
| Marshmallow | *Althaea officinalis* | 1.50% |
| Tormentil | *Potentilla erecta* | 0.50% |
| Dandelion | *Taraxacum officinale* | 3.25% |


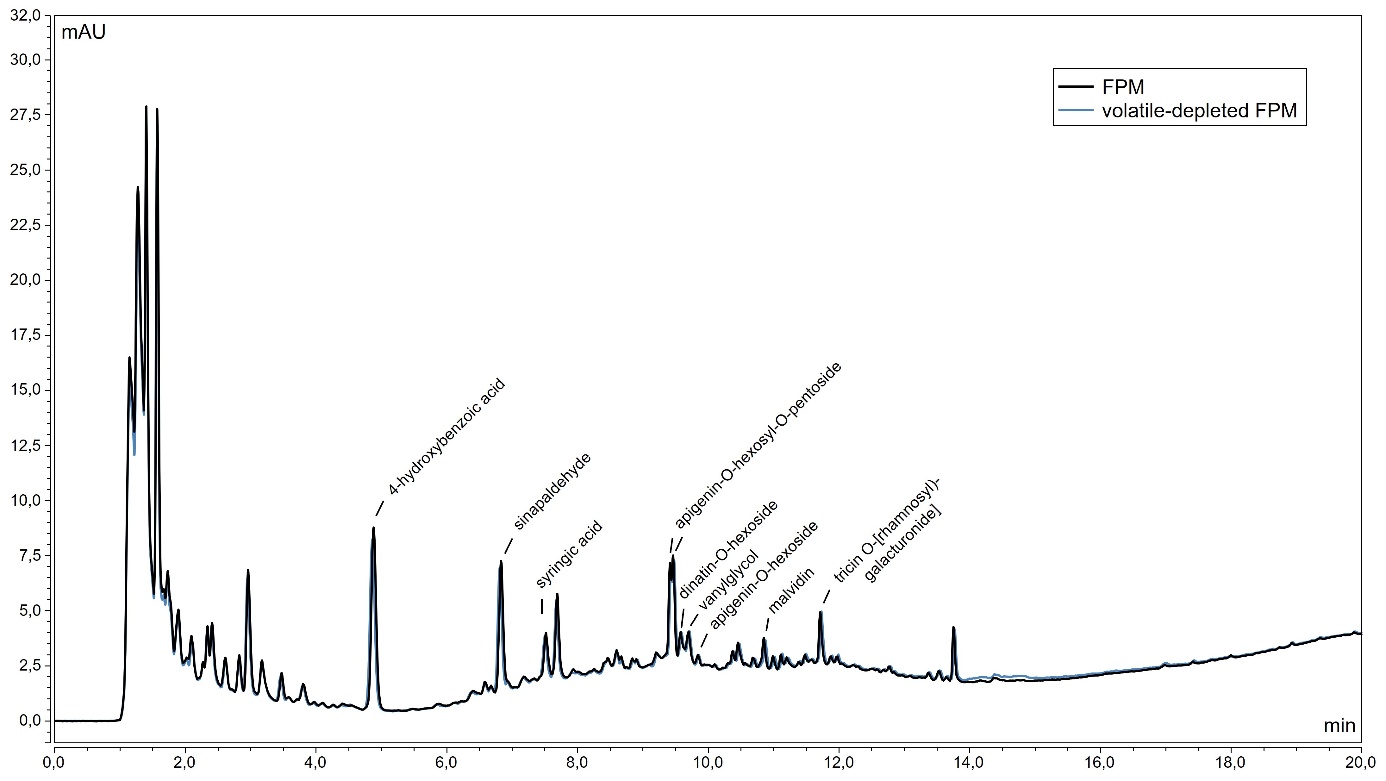


**Fig. S1**: Overlay of HPLC-DAD chromatograms of FPM and volatile-depleted FPM. Injection volume was 1 µL each and detection wavelength was set to 260 nm. Compounds were tentatively identified based on the HR-MS measurements as listed in Table S2.

**Table S2**: Overview of the tentatively identified compounds in FPM through HPLC-MS.

| **Retention time (min)** | **UV _max_** | **[M+H]^+^** | **MW** | **Formula** | **Tentative identification** |
| --- | --- | --- | --- | --- | --- |
| 1.53 | 241 | 274.0920 | 273.0847 | C12H11N5O3 | - |
| 1.64 | 242 | 151.0755 | 150.0682 | C10H14O | - |
| 2.48 | 220 | 129.0545 | 128.0473 | C6H8O3 | - |
| 2.6 | 225 | 127.0390 | 126.0317 | C6H6O3 | 5-hydroxymethylfurfural |
| 4.92 | 256 | 139.0388 | 138.0315 | C7H6O3 | 4-hydroxybenzoic acid |
| 5.41 | 231/297 | 339.1074 | 338.1001 | C16H18O8 | coumaroylquinic acid |
| 6.64 | 261/292 | 209.0808 | 208.0735 | C11H12O4 | sinapaldehyde / 3,4-dimethoxycinnamic acid |
| 7.34 | 227/275 | 199.0598 | 198.0526 | C9H10O5 | syringic acid / ethyl gallate |
| 7.86 | 231/286 | 177.0544 | 176.0471 | C8H10O3 | - |
| 8.79 | 272/331 | 565.1550 | 564.1477 | C26H28O14 | apigenin O-hexosyl-O-pentoside |
| 8.85 | 272/331 | 565.1550 | 564.1477 | C26H28O14 | apigenin O-hexosyl-O-pentoside |
| 9.07 | 272/335 | 463.1230 | 462.1157 | C22H22O11 | dinatin-O-hexoside |
| 9.46 | 275 | 185.0807 | 184.0734 | C9H12O4 | vanylglycol / trimethoxyphenol |
| 9.71 | 271/321 | 433.1128 | 432.1055 | C21H20O10 | apigenin O-hexoside |
| 9.81 | 270/328 | 331.0811 | 331.0811 | C17H15O7+ | malvidin |
| 10.48 | 275/330 | 233.0806 | 232.0733 | C12H12O4 | eugenitin |
| 10.98 | 270/335 | 507.1131 | 506.1058 | C23H22O13 | acetylhyperin |
| 11.09 | 268/351 | 653.1710 | 652.1637 | C29H32O17 | tricin O-[rhamnosyl-galacturonide] |
| 13.09 | 230/266 | 207.1014 | 206.0938 | C12H14O3 | - |
| UV absorbance maxima (UV max), protonated molecular ion peaks ([M+H]+), molecular weights (MW) | | | | | |
